# High School Football and Risk for Depression and Suicidality in Adulthood: Findings From a National Longitudinal Study

**DOI:** 10.3389/fneur.2021.812604

**Published:** 2022-02-09

**Authors:** Grant L. Iverson, Douglas P. Terry

**Affiliations:** ^1^Department of Physical Medicine and Rehabilitation, Harvard Medical School, Boston, MA, United States; ^2^Department of Physical Medicine and Rehabilitation, Spaulding Rehabilitation Hospital, Charlestown, MA, United States; ^3^Spaulding Research Institute, Charlestown, MA, United States; ^4^MassGeneral Hospital for Children Sports Concussion Program, Boston, MA, United States; ^5^Home Base, A Red Sox Foundation and Massachusetts General Hospital Program, Charlestown, MA, United States; ^6^Vanderbilt Sports Concussion Center, Department of Neurological Surgery, Vanderbilt University Medical Center, Nashville, TN, United States

**Keywords:** suicide, concussion, head injury, traumatic brain injury, football, depression, mental health, anxiety

## Abstract

**Background:**

There is growing public concern regarding the potential long-term effects of playing football on brain health, specifically that playing football before and during high school might cause damage to the brain that manifests years or decades later as depression or suicidality. This study examined if playing high school football was associated with increased lifetime risk for depression, suicidality over the past year, or depressed mood in the past week in men aged between their middle 30 s to early 40 s.

**Methods:**

Publicly available data from the National Longitudinal Study of Adolescent to Adult Health were analyzed. This longitudinal, prospective cohort study sampled nationally representative U.S. youth starting in 1994–1995 (Wave I) and most recently in 2016–2018 (Wave V). A total of 3,147 boys participated in Wave I (median age = 15), of whom 1,805 were re-assessed during Wave V (median age = 38).

**Results:**

Of the 1,762 men included in the study, 307 (17.4%) men reported being diagnosed with depression and 275 (15.6%) reported being diagnosed with an anxiety disorder or panic disorder at some point in their life. When comparing men who played high school football to those who did not, there were no differences in the proportions of the sample who had a lifetime diagnosis of depression, lifetime diagnosis of anxiety/panic disorders, suicidal ideation in the past year, psychological counseling in the past year, or current depressed mood. However, men who received psychological counseling and/or experienced suicidal ideation during adolescence were significantly more likely to report a lifetime history of depression, suicidal ideation in the past year, and current depressed mood.

**Conclusion:**

Individuals who reported playing football during adolescence did not have an increased risk of depression or suicidal ideation when they were in their middle 30 s to early 40 s, but mental health problems during adolescence were associated with an increased risk for psychological health difficulties more than 20 years later.

## Introduction

American football has been popular for generations, and more than one million youth participate at the high school level annually (1). In recent years, there has been significant public concern regarding possible long-term effects on brain health associated with playing football. In one study, 54% of adults reported that they would not allow their child to play youth football (2). A central concern is that playing youth football, before and during high school, might cause damage to the brain that manifests clinically years or decades later as depression, suicidality, cognitive impairment, or neurological disease. It has been documented that playing youth football results in a large exposure to blows to the head, with the average player sustaining hundreds of head impacts per season (3, 4). Football players also are more likely to experience concussions than youth participating in other sports (5, 6). Researchers have reported that playing a single season of high school football is associated with changes in the brain measurable with structural and functional experimental neuroimaging (7, 8), as well as changes in cognition (9) and other clinical metrics, though results have been mixed Walter et al. (10). Concerns about long-term brain health have likely been fueled by extensive media coverage of research relating to chronic traumatic encephalopathy (CTE) in former National Football League (NFL) players (11, 12). Some researchers have asserted that depression and suicidality are clinical features of CTE (13–19), although multiple reviews of the literature have not identified suicide to be a clinical feature (20–25).

A personal history of multiple concussions has been associated with increased risk for later-in-life depression, or symptoms of depression, in former National Collegiate Athletic Association (NCAA) players (26) and retired NFL players (27–31). Survey studies reveal that some retired NFL players experience symptoms of depression (27), and these symptoms have been associated with chronic pain (32), pain catastrophizing (33), current opioid use (34), decline in physical functioning (27), and insufficient sleep (28). However, retired NFL players are at lower risk for suicide, not greater risk, than men from the general population (25, 35, 36).

Several studies have examined whether participating in high school football is associated with later in life psychological health problems. Three research teams used The National Longitudinal Study of Adolescent to Adult Health database to examine whether boys who played high school football are more likely to have mental health problems during *early adulthood* (i.e., late 20 s) (37–39). Those researchers reported that individuals who played high school football did not report greater lifetime rates of anxiety (38) or depression (37–39), suicidal ideation within the past year (37–39), or current symptoms of depression (i.e., within the past seven days) (38). An obvious limitation of those studies is that there were only a few years between high school graduation and follow-up—and having a longer interval would be important to better assess whether football confers risk for mental health problems in early or middle adulthood. A survey of more than 400 *middle-aged* men from the United States, ages 35–55, revealed that those who played high school football were not more likely to have a lifetime history of treatment for mental health problems, nor did they have higher rates of depression, anxiety, and anger in the preceding year, compared to men who did not play high school football (40), although these participants were recruited from an online platform and may not be representative of the general population. Two research teams examining data from *older adult* men participating in the Wisconsin Longitudinal Study reported no association between playing high school football and later in life psychological health or self-rated physical health at the average age of 65 (41, 42).

Between 2016 and 2018, another wave of interviews were completed as part of the National Longitudinal Study of Adolescent to Adult Health, when the participants were in their middle 30 s to early 40 s—and the data has been made publicly available. The purpose of this study was to determine whether playing high school football is associated with increased lifetime risk for depression, suicidality over the past year, or depressed mood in the past week in men who are in their middle 30 s to early 40 s. This is an important time period in life to study given that the possibility of experiencing depression or suicidality at some point during one's life increases over time, and the prior studies that found a relationship between football and depression examined former collegiate and professional players who were in their mid-to-late 30 s (26) and older (27, 28). Moreover, according to the recent consensus criteria for traumatic encephalopathy syndrome (43), former high school football players are considered to be at risk decades after exposure and depression and suicidality are reported to be “supportive” features that are often present in traumatic encephalopathy syndrome (although not part of the diagnostic criteria for the syndrome). We identified possible risk factors for suicidality during adolescence and examined them as predictors for suicidality more than two decades later. We hypothesized that having mental health difficulties during adolescence, especially suicidality, would be an important predictor of experiencing suicidality later in life, a hypothesis consistent with findings from when these participants were interviewed 10 years prior, during Wave IV (39). In contrast, as the primary focus of the study, we hypothesized that playing football during adolescence would not be significantly related to depression and suicidality in these men, consistent with findings from when they were interviewed ~10 years prior (37–39).

## Materials and Methods

### Participants

Public-use data collected via the National Longitudinal Study of Adolescent to Adult Health (i.e., “Add Health”) was used for this study (44). This longitudinal study started during the 1994–1995 school year when the participants were adolescents (i.e., grades 7–12; Wave I). The sample was collected using systematic sampling methods from 80 high schools and 50 middle schools to ensure that the demographics of participants were representative of U.S. schools regarding size, type, ethnicity, region, and urbanicity. During the first Wave, 20,745 youth completed the full Add Health study, and in the public use version of the database that was used in this specific study, Wave I data was available for 6,504 participants. Some of these individuals completed follow-up interviews in 2016–2018 when the participants were ~34–44 years old (Wave V, *n* = 4,196) (45). Separate databases from Waves I and V were merged using participant identification numbers so that data could be examined longitudinally. From this merged database, participants were included in this study if they identified as male in the Wave I database (variable “BIO_SEX; ”*n* = 3,147 total boys at Wave I) and completed the main outcome measures related to depression and suicidality at the Wave V assessment (variables H5ID6G and H5MN8; *n* = 1,805; 1,342 lost to follow-up).

### Survey Questions

Football exposure was assessed during the Wave I assessment with the question “Are you participating/Do you plan to participate in the following clubs, organizations and teams (check all that apply): Football” (question S44A21). All questions relating to participation in specific sports were asked in this manner—and thus it is not possible to determine with certainty, whether all the boys who reported yes actually played football. Related to mental health at Waves I and V, we extracted data related to a lifetime history of depression (Wave V question H5ID6G: “Has a doctor, nurse or other health care provider ever told you that you have or had depression?”), lifetime history of anxiety (Wave V question H5ID6I: “Has a doctor, nurse, or other health care provider ever told you that you have or had anxiety or panic disorder?”), suicidal ideation (Wave I question H1SU1 and Wave V question H5MN8: “During the past 12 months, have you ever seriously thought about committing suicide?”), psychological treatment (Wave I question H1HS3 and Wave V question H5ID13: “In the past 12 months, have you received psychological or emotional counseling?”), and current depressed mood (question H5SS0B “During the past 7 days, I felt depressed;” response options: never or rarely, sometimes, a lot of the time, most of the time or all the time).

### Statistical Analyses

SPSS version 28.0 was used for all analyses. The threshold for statistical significance was set at *p* < 0.05. Descriptive statistics were used to summarize characteristics from the sample. Chi-square tests of association were used to examine whether football participation during adolescence was associated with the proportion of the sample who endorsed a lifetime history of depression, suicidal ideation over the past year, and depressed mood over the past 7 days.

## Results

The publicly available database includes 3,147 boys who participated in Wave I of the study during their adolescence. Of these, 1,805 men participated in the Wave V follow-up ~21–24 years after the initial study (57.3%). A small number of participants were excluded because they did not provide answers to the main outcome questions (i.e., lifetime history of depression and/or suicidal thoughts in the past year, *n* = 43). The final sample included 1,762 men. Demographics for the final sample are included in Table 1. The sample was, on average, 38 years old at the Wave V assessment. Data collection for Wave V occurred in 2016 (26.3%), 2017 (50.3%), and 2018 (23.3%).

**Table 1:** Sample demographics.

	**Entire sample** **(*N* = 1,762)**	**Football** **(*n* = 369)**	**No football** **(*n* = 952)**
**Age at wave V**			
Mean	38.03	37.65	38.04
Standard deviation	1.95	1.80	1.93
Median	38	38	38
Interquartile range	36–40	36–39	36–40
Range	34–44	34–43	34–43
**Age at Wave I**			
Mean	14.95	14.72	15.03
Standard deviation	1.74	1.71	1.75
Median	15	15	15
Interquartile range	14–16	13–16	14–16
Range	11–19	12–19	11–19
**Race**			
White	72.4%	64%	75.1%
Black/African American	18.2%	27.4%	16.0%
American Indian/Native American	1.2%	1.4%	0.7%
Asian/pacific islander	3.6%	3.8%	3.9%
Other	4.5%	3.5%	4.3%
Don't know	0.1%	0%	0%
**Education**			
Some high school	5.6%	3.3%	4.8%
High school diploma/general equivalency diploma	19.9%	20.1%	17.7%
Some college/vocational school/associate's degree	39.3%	36.4%	40.3%
Bachelor's degree	19.7%	22.2%	19.9%
More than bachelor's degree	15.5%	18.1%	17.3%

### Football in High School and Mental Health During Adulthood

**Figure 1:**
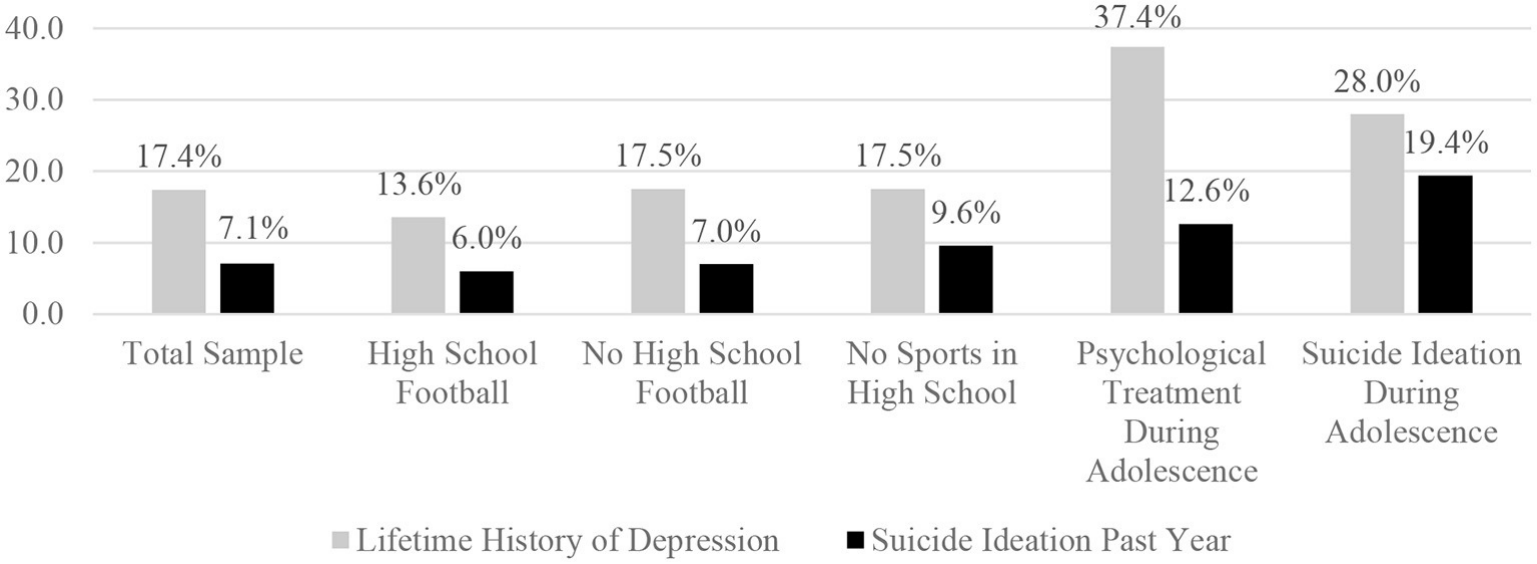
Percentages of men who reported experiencing suicide ideation in the past year during the Wave V interview. The percentages are based on the number of men who reported experiencing suicidal ideation during the past year. The total sample included 1,762 men who were interviewed at, on average, age 38 during the Wave V interview. The sample sizes for the subgroups are as follows: high school football *n* = 369; no high school football *n* = 952; no sports in high school *n* = 460; psychological treatment during adolescence *n* = 174; and suicide ideation during adolescence *n* = 186.

During the Wave V assessment, 307 (17.4%) men reported being diagnosed with depression at some point in their life, 275 (15.6%) being diagnosed with an anxiety disorder or panic disorder at some point in their life, 211 (12.0%) having received psychological or emotional counseling in the past 12 months, 125 (7.1%) reported seriously thinking about suicide in the past year, and 101 (5.8%) reported feeling depressed in the previous week (i.e., “a lot of the time” or “most of the time or all of the time” over the past 7 days). Examining responses the participants gave during the Wave I assessment when they were adolescents, 369 (20.9%) reported playing (or intending to play) football in high school and 952 (54.0%) reported not intending to play football in high school. Of note, 441 participants (25% of the sample) did not answer this question and were excluded from analyses pertaining to football participation. Participants who played football, compared to participants who did not, had similar rates of (i) being diagnosed with depression at some point in their life [13.6 vs. 17.5%; $\chi_{\left(1\right)}^{2}$ = 3.09, *p* = 0.08, OR = 0.74 95% CI = 0.52–1.04; see Figure 1], (ii) being diagnosed with an anxiety disorder or panic disorder at some point in their life [13.4 vs. 16.1%, $\chi_{\left(1\right)}^{2}$ = 1.53, *p* = 0.22, OR = 0.80, 95% CI = 0.59–1.14], (iii) having received psychological or emotional counseling in the past 12 months [10.4 vs. 11.6%, $\chi_{\left(1\right)}^{2}$ = 0.37, *p* = 0.54, OR = 0.89, 95% CI = 0.60–1.31], (iv) suicidal ideation in the past year [6.0 vs. 7.0%; $\chi_{\left(1\right)}^{2}$ = 0.49, *p* = 0.48, OR = 0.84, 95% CI = 0.51–1.38], and (v) feeling depressed in the past 7 days [4.1 vs. 6.2%; $\chi_{\left(1\right)}^{2}$ = 2.12, *p* = 0.15, OR = 0.65, 95% CI = 0.37–1.17].

### Mental Health in Adolescence and Depression and Suicide Ideation in Adulthood

During Wave I, there were 174 boys (9.9%) who reported undergoing psychological counseling in the past year while in high school. During the Wave V interview, ~24 years later, those individuals who underwent psychological counseling during adolescence were much more likely to report (i) a lifetime history of depression [37.4 vs. 15.3%, $\chi_{\left(1\right)}^{2}$ = 53.17, *p* < 0.001, OR = 3.31, 95% CI = 2.37–4.64], (ii) a lifetime history of anxiety disorder or panic disorder [27.0 vs. 14.4%, $\chi_{\left(1\right)}^{2}$ = 18.88, *p* < 0.001, OR = 2.20, 95% CI = 1.53–3.16], (iii) having received psychological or emotional counseling in the past 12 months [21.3 vs. 11.0%, $\chi_{\left(1\right)}^{2}$ = 14.48, *p* < 0.001, OR = 2.18, 95% CI = 1.47–3.24], (iv) suicidal ideation in the past year [12.6 vs. 6.4%, $\chi_{\left(1\right)}^{2}$ = 9.24, *p* = 0.002, OR = 2.11, 95% CI = 1.29–3.44], and (v) current depression [11.6 vs. 5.1%, $\chi_{\left(1\right)}^{2}$ = 11.77, *p* < 0.001, OR = 2.41, 95% CI = 1.43–4.04], compared to those who did not.

When interviewed during adolescence, 186 boys (10.6%) endorsed thoughts of suicide in the past year. At the follow-up assessment, ~24 years later, those men who reported suicide ideation during adolescence, compared to those who did not, were more likely to report (i) a lifetime history of depression [28.0 vs. 16.0%, $\chi_{\left(1\right)}^{2}$ = 16.54, *p* < 0.001, OR = 2.03, 95% CI = 1.44–2.88], (ii) having received psychological or emotional counseling in the past 12 months [21.5 vs. 10.8%, $\chi_{\left(1\right)}^{2}$ = 17.95, *p* < 0.001, OR = 2.26, 95% CI = 1.54–3.31], (iii) suicide ideation in the past year [19.4 vs. 5.6%, $\chi_{\left(1\right)}^{2}$ = 48.18, *p* < 0.001, OR = 4.06, 95% CI = 2.66–6.20], and (iv) feeling depressed within the past 7 days [13.0 vs. 4.8%, $\chi_{\left(1\right)}^{2}$ = 20.79, *p* < 0.001, OR = 2.97, 95% CI = 1.82–4.84]. Those who reported suicidal ideation during adolescence had a higher lifetime history of anxiety disorder or panic disorder, but this result was not statistically significant [20.4 vs. 15.0%, $\chi_{\left(1\right)}^{2}$ = 3.78, *p* = 0.052, OR = 1.46, 95% CI = 0.995–2.14].

## Discussion

The present study found no association between playing high school football and lifetime history of anxiety or depression more than 20 years later. Moreover, men in their middle 30 s and early 40 s, who played high school football, did not report greater rates of receiving mental health treatment in the past year, experiencing suicidal ideation in the past year, or experiencing current symptoms of depression. In contrast, men who underwent mental health treatment during their youth or experienced suicidal ideation were significantly more likely to undergo mental health treatment and/or experience suicidal ideation more than 20 years later.

The National Longitudinal Study of Adolescent to Adult Health has been used by researchers to examine whether playing high school football is associated with mental health problems in early to middle adulthood. The longitudinal study began in 1994, and the most recent wave of data collection was completed 24 years later, in 2018. Including the present study, four studies have relied on Wave III (2001–2001; median age of 22, IQR = 20–23), Wave IV (2007–2009; median age of 29, IQR = 28–31), and Wave V (2016–2018, median age of 38, IQR = 36–40) data from this study. Researchers have reported that men who played high school football, when surveyed later, at the average age of 29, did not report a greater lifetime history of anxiety (38) or depression (37–39), a greater rate of receiving mental health treatment in the past year (39), or current symptoms of depression (i.e., within the past seven days) (39). Those were the same findings in the present study, for all of those variables, a decade later, when they were an average age of 38. These longitudinal data also reveal that young men who played high school football were not more likely to report suicidal ideation within the past year at an average age of 22 (39), 29 (37–39), or 38 (present study). In sum, none of these studies found an effect between high school football participation and higher rates of mental health problems or suicidality at the follow-up time point.

Three additional studies have reported that men who played high school football did not report greater mental health problems during middle age (40) or older adulthood (41, 42). A recent post-mortem study, from a brain donation program, reported that there was no statistically significant difference in the proportions of suicide as a manner of death among those men with a personal history of playing football compared to men who did not play football or who did not play sports (46). Moreover, those who played football were significantly less likely to have a lifetime history of a suicide attempt (46). In aggregate, the above-mentioned studies suggest that men who played high school football are not at greater risk for suicidality in early adulthood, during middle age, or as older adults.

### Implications for Traumatic Encephalopathy Syndrome

Traumatic Encephalopathy Syndrome (TES) (43, 47) is conceptualized as the clinical syndrome that *might* be associated with chronic traumatic encephalopathy (CTE) neuropathologic change (48). The National Institute of Neurological Disorders and Stroke (NINDS) Consensus Diagnostic Criteria for TES were published in 2021 (43). According to these criteria, everyone who played organized football for five or more years, with at least 2 years at the high school level, is at risk for TES. Depression, anxiety, and suicidality are not considered core diagnostic features of TES, but they are considered to be supportive features that are believed to frequently occur. The cumulative literature published to date (described above), including the present study, does not support a belief that a large percentage of former high school football players will develop TES with associated serious mental health problems including depression and suicidality.

### Limitations

There are several important limitations related to this study. First, all data were self-reported and could be affected by reporting biases. Second, as noted in the Methods section, the football exposure variable assessed if the students were currently participating in football or planned to participate in football; this was the format of the question used for all sports because the survey would have been conducted at different points in time during the school year. There may be some students who planned to play football but ultimately did not, as well as students who did not plan to play football but nonetheless joined the team. Third, several pieces of information about the participants' football/contact sport exposure were not collected (e.g., age of first exposure, length of career, position, and number of concussions), limiting the ability to examine these data in a more continuous manner based on one's cumulative football or repetitive neurotrauma exposure. Lastly, this study examined men who played, or planned to play, high school football and may not be applicable to those who played other contact or collision sports—and the results of this study are not applicable to women.

### Sports and Exercise: Protective Factors

It is possible that playing high school football confers “risk” for future mental health problems through exposure to concussions, repetitive mild neurotrauma, and/or orthopedic injuries leading to later in life chronic pain—while at the same time being “protective,” through various resistance and resilience factors. There are many general health benefits to participating in sports, such as lower rates of obesity (49), better cardiovascular fitness (50), and greater lean muscle mass (51). There are diverse psychosocial benefits (52) such as greater social connectedness (51, 53, 54), and greater self-confidence (54) and self-esteem (55, 56). Not exercising or participating in sports is associated with lower life satisfaction in high school students (57). There are mental health benefits, such as less anxiety and other psychological health problems (52), and lower rates of depression (58, 59) and suicide (60–62). Involvement in sports and exercise might also be associated with positive differences in brain neurobiology (63) and better cognitive functioning (63–65), at least in some studies.

If youth who participate in sports are more likely to exercise during adulthood (66, 67), then this would confer a number of protective health benefits across the lifespan. Greater physical activity and exercise (especially with dietary modifications) are associated with reduced risk for obesity, hypertension, diabetes, and metabolic syndrome in adults (68–70), better sleep (71–73), and better mental health (74, 75). Moreover, there is a strong rationale for using exercise and physical activity for health promotion, disease prevention, and treatment in older adults (76).

## Conclusions

With an annual participation rate of more than 1 million per year (1), it is reasonable to assume that many millions of men, over the past few decades, in the United States participated in high school football. It is important to study these men to determine if they experience later-in-life mental health problems that might be associated with repetitive neurotrauma, chronic pain, or other health issues. There are several studies to date suggesting that men who played high school football are not at greater risk for depression or suicidality in early adulthood (37–39), during middle age (40), or as older adults (41, 42). The results of this study are consistent with prior studies that do not find an association between high school football exposure and later mental health problems, including depression, anxiety, and suicidality.

Without question, however, some men who played high school football will later experience depression, suicidality, or both—just like other men in the general population. Suicidality is a clinical feature of depression, and when people are depressed some risk factors for completing suicide include a family history of a psychiatric disorder, previous attempted suicide, more severe depression, hopelessness, comorbid anxiety, and misuse of alcohol or drugs (77). High risk patterns of thinking associated with suicidality include hopelessness (78, 79), perceived burdensomeness (80), and mental pain (81). Related to family history of depression there are several genetic and epigenetic factors that likely influence the clinical manifestation of depression and suicidality (82, 83). In older adults, suicidality is associated with a broad range of medical problems including malignant diseases, neurological disorders, arthritis, chronic obstructive pulmonary disease, and liver disease (84). Moreover, in older adults, moderate to severe pain is associated with increased risk for suicidal ideation and attempts, and pain is a much stronger predictor for suicide in men than in women (85). It is important to appreciate that there are evidence-based treatments for depression and suicidality that can greatly reduce suffering and improve quality of life in these men.

## Data Availability Statement

Publicly available datasets were analyzed in this study. This data can be found here: Add Health, https://addhealth.cpc.unc.edu/data/#public-use.

## Ethics Statement

Add Health participants provided written informed consent for participation in accordance with the University of North Carolina School of Public Health Institutional Review Board guidelines. The data used in this manuscript were de-identified and is publicly available. No additional approvals were necessary in accordance with the local legislation and institutional requirements.

## Disclosure

GI serves as a scientific advisor for NanoDX®, Sway Operations, LLC, and Highmark, Inc. He has a clinical and consulting practice in forensic neuropsychology, including expert testimony, involving individuals who have sustained mild TBIs (including former athletes), and on the topic of suicide. He has received research funding from several test publishing companies, including ImPACT Applications, Inc., CNS Vital Signs, and Psychological Assessment Resources (PAR, Inc.). He has received research funding as a principal investigator from the National Football League, and subcontract grant funding as a collaborator from the Harvard Integrated Program to Protect and Improve the Health of National Football League Players Association Members. He also has research funding from the Wounded Warrior Project®. DT has served as a consultant for REACT Neuro, Inc. He has a consulting practice in forensic neuropsychology, including expert testimony, involving individuals who have sustained mild TBIs (including former athletes).

## Author Contributions

GI conceptualized the study, conducted the literature review, helped conceptualized the statistical analyses, wrote portions of the manuscript, and agrees to be accountable for the content of the work. DT helped conceptualize the study, the statistical analyses, conducted the statistical analyses, wrote portions of the manuscript, and agrees to be accountable for the content of the work. Both authors contributed to the article and approved the submitted version.

## Funding

GI acknowledges unrestricted philanthropic support from ImPACT Applications, Inc., the Mooney-Reed Charitable Foundation, the Boston Bolts, the National Rugby League, and the Spaulding Research Institute. These entities were not involved in the study design, collection, analysis, interpretation of data, the writing of this article or the decision to submit it for publication.

## Conflict of Interest

GI has a clinical and consulting practice in forensic neuropsychology, including expert testimony, involving individuals who have sustained mild TBIs (including former athletes), and on the topic of suicide. The authors declare that the research was conducted in the absence of any commercial relationships that could be construed as a potential conflict of interest.

## Publisher's Note

All claims expressed in this article are solely those of the authors and do not necessarily represent those of their affiliated organizations, or those of the publisher, the editors and the reviewers. Any product that may be evaluated in this article, or claim that may be made by its manufacturer, is not guaranteed or endorsed by the publisher.
